# Ultraviolet Photoactivated Room Temperature NO_2_ Gas Sensor of ZnO Hemitubes and Nanotubes Covered with TiO_2_ Nanoparticles

**DOI:** 10.3390/nano10030462

**Published:** 2020-03-04

**Authors:** Hee-Jung Choi, Soon-Hwan Kwon, Won-Seok Lee, Kwang-Gyun Im, Tae-Hyun Kim, Beom-Rae Noh, Sunghoon Park, Semi Oh, Kyoung-Kook Kim

**Affiliations:** 1 Department of Advanced Convergence Technology, and Research Institute of Advanced Convergence Technology, Korea Polytechnic University, 237 Sangidaehak-ro, Siheung-si 15073, Korea; gmlwjd0889@kpu.ac.kr (H.-J.C.); canwkd21@kpu.ac.kr (S.-H.K.); dldnjstjr37@kpu.ac.kr (W.-S.L.); ksdd67@kpu.ac.kr (T.-H.K.); bruno5015@kpu.ac.kr (B.-R.N.); 2 Department of Nano & Semiconductor engineering, Korea Polytechnic University, 237 Sangidaehak-ro, Siheung-si 15073, Korea; rhkdrbs87@kpu.ac.kr; 3 Department of Software Convergence, and 1101B Gwanggeto-Bd, Sejong University, Neungdong-ro, Gwangjin-gu, Seoul 05006, Korea; s.park@sejong.ac.kr; 4 Department of Electrical Engineering and Computer Science, University of Michigan, 1301 Beal Avenue, Ann Arbor, Michigan 48109, USA

**Keywords:** room temperature, NO_2_ gas sensor, ZnO hemitube, TiO_2_ nanoparticle, UV emitter

## Abstract

Prolonged exposure to NO_2_ can cause lung tissue inflammation, bronchiolitis fibrosa obliterans, and silo filler’s disease. In recent years, nanostructured semiconducting metal oxides have been widely used to fabricate gas sensors because of their unique structure and surface-to-volume ratio compared to layered materials. In particular, the different morphologies of ZnO-based nanostructures significantly affect the detection property of NO_2_ gas sensors. However, because of the large interaction energy of chemisorption (1–10 eV), metal oxide-based gas sensors are typically operated above 100 °C, overcoming the energy limits to attain high sensitivity and fast reaction. High operating temperature negatively affects the reliability and durability of semiconductor-based sensors; at high temperature, the diffusion and sintering effects at the metal oxide grain boundaries are major factors causing undesirable long-term drift problems and preventing stability improvements. Therefore, we demonstrate NO_2_ gas sensors consisting of ZnO hemitubes (HTs) and nanotubes (NTs) covered with TiO_2_ nanoparticles (NPs). To operate the gas sensor at room temperature (RT), we measured the gas-sensing properties with ultraviolet illumination onto the active region of the gas sensor for photoactivation instead of conventional thermal activation by heating. The performance of these gas sensors was enhanced by the change of barrier potential at the ZnO/TiO_2_ interfaces, and their depletion layer was expanded by the NPs formation. The gas sensor based on ZnO HTs showed 1.2 times higher detection property than those consisting of ZnO NTs at the 25 ppm NO_2_ gas.

## 1. Introduction

NO_2_ is a pungent red-brown oxidizing gas that is released into the atmosphere from both natural sources and human activities. Prolonged exposure to this gas can cause lung tissue inflammation, bronchiolitis fibrosa obliterans, and silo filler’s disease. In plants, exposure to several ppm of NO_2_ results in inadequate chlorophyll synthesis, causing chlorosis and other plant tissue breakdowns. Furthermore, NO_2_ emitted by supersonic jets causes the destruction of the ozone layer present in the stratosphere, which absorbs the harmful ultraviolet (UV) radiation coming from the sun [1,2,3].

In recent years, nanostructured semiconducting metal oxides such as SnO_2_, ZnO, TiO_2_, and CuO have been widely used to fabricate gas sensors because of their unique structure and surface-to-volume ratio compared to layered materials [4,5,6]. Among them, ZnO is largely utilized for the realization of various nanostructures, including nanorods (NRs), nanotubes (NTs), nanowires (NWs), nanowalls, and nanoflowers [7,8,9,10]. In particular, the different morphologies of ZnO NRs significantly affect the detection property of NO_2_ gas sensors [11]; moreover, ZnO NRs-based sensors have a very low detection limit (10 ppm) for NO_2_ gas at 250 °C, along with short response and recovery times [12]. TiO_2_ is another very promising material for NO_2_ gas detection because of its high temperature stability, harsh environment tolerance, and catalytic properties [13]. Therefore, combined ZnO/TiO_2_ nanostructures can achieve better physical and chemical properties than of the corresponding individual ones because of the modification of their electronic states [14,15].

The quality of a metal oxide-based gas sensor is represented by its sensitivity, selectivity, and stability. Adding a second component can easily improve both sensitivity and selectivity; however, without an effective solution, stability is still limited [16]. The working principle of these gas sensors is based on the surface chemical reactions that interfere with the free carrier density of their constituent metal oxide. This allows the detection of changes in the surrounding environment by measuring the electrical characteristics of the sensor. As a result of the large interaction energy of chemisorption (1–10 eV), metal oxide-based gas sensors are typically operated above 100 °C, overcoming the energy limits to attain high sensitivity and fast reaction [17]. However, high operating temperature negatively affects the reliability and durability of semiconductor-based sensors; at high temperature, the diffusion and sintering effects at the metal oxide grain boundaries are major factors causing undesirable long-term drift problems and preventing stability improvements [18].

In this study, we fabricated gas sensors consisting of ZnO hemitubes (HTs) and NTs covered with TiO_2_ nanoparticles (NPs) to achieve a high response and quick response/recovery times for NO_2_ gas detection. The oxygen-related gas-sensing mechanism involves the absorption of oxygen molecules on the oxide surface to generate chemisorbed oxygen species (O_2_^−^, O^2−^, O^−^) by capturing electrons from the conductance band of semiconductor material, making the oxide surface highly resistive. The oxide is exposed to the traces of the introduced reductive gas. In reacting with the oxygen species at the oxide surface, the reductive gas reduces the concentration of oxygen species at this surface and thereby increases the electron concentration [19]. Therefore, ZnO NTs are able to provide large sensitivity because of their large length-to-diameter ratio and surface-to-volume ratio than other nanostructures. There are several methods to realize ZnO NTs such as the temperature change etching process, wet chemical etching process, and removing some materials by heating [9,13,20]. We fabricated the ZnO tube shapes by ZnO deposition thickness on the polyvinylpyrrolidone (PVP) NWs and removing PVP NWs. In addition, to solve the problem of sensing material stability at high temperature and obtain high energy efficiency, we used the UV emitter (365 nm) for photoactivation rather than a high-temperature heating process for increasing NO_2_ gas detection at room temperature (RT).

## 2. Materials and Methods 

### 2.1. PVP NWs

A 300 nm SiO_2_ insulating layer was deposited on a Si substrate by plasma-enhanced chemical vapor deposition. Then, PVP NWs were synthesized by electrospinning onto the substrate. The electrospinning solution was obtained by mixing ethanol (2.5 mL), dimethylformamide (2.5 mL), and PVP (0.75 g). Subsequently, it was blended by dissolving PVP in the solvent under stirring at 60 °C for 12 h. The PVP solution was placed in a glass syringe equipped with a 21-gauge stainless steel needle; the distance between the needle tip and an Al flat-plate collector was fixed at 20 cm. A positive voltage of 18 kV was applied to the needle, while the collector was grounded. At the same time, an additional negative voltage of 15 kV was applied to the Al plate to accelerate the electrospinning process. The solution feed rate was adjusted at 0.01 mL/min and kept constant by a syringe pump. The as-obtained PVP NWs were uniformly distributed onto the substrate, which was placed on the Al metal collector. A ring-type collector was tested to fabricate NWs in a random network form.

### 2.2. Gas Sensor Materials Fabrication

**Figure 1:**
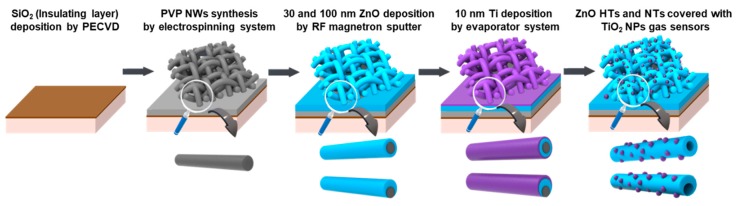
Fabrication procedure for gas sensors consisting of ZnO hemitubes (HTs) and nanotubes (NTs) covered with TiO_2_ nanoparticles (NPs).

Figure 1 shows the fabrication procedure of ZnO HTs and ZnO NTs covered with a TiO_2_ NPs gas sensor. To evaluate the influence of the ZnO layer thickness on the structural and sensing property, the 30 nm and 100 nm ZnO layers were deposited on the PVP NWs through radio-frequency sputtering. The base pressure and working pressure for the ZnO deposition were 2.0 × 10^-^^7^ Torr and 3 mTorr, respectively, in argon ambient at a sputtering power of 60 W and a deposition rate of 0.0625 nm/sec. To fabricate TiO_2_ NPs, a 10 nm Ti layer was deposited onto the ZnO layers by an electron-beam evaporator; the base pressure was 1.0 × 10^-7^ Torr, the power was 5.0%, and the deposition rate was 0.2 Å/sec. To remove the PVP NWs and form TiO_2_ NPs, the annealing process on the samples was performed at 800 °C in O_2_ ambient for 1 h. By removing the PVP NWs as high-temperature heat, ZnO HTs and NTs were self-formed, and the Ti layer was turned into TiO_2_ NPs by agglomeration on the ZnO surface. 

### 2.3. Gas Sensor Materials Characterization

The morphological and structural properties of the synthesized samples were characterized by using a field emission-scanning electron microscopy (FE-SEM) (Hitachi S-4300, Hitachi Ltd., TYO, JP) and a transmission electron microscopy (TEM) (JEOL 2100F, JEOL Ltd., TYO, JP) operated at 10 kV and 300 kV, respectively. In addition, elemental mapping was conducted with an energy-dispersive spectroscopy (EDS) (Oxford Inca x-sight, Oxford Instruments Inc., Oxon, UK).

### 2.4. Gas Sensor Characterization

We fabricated interdigital electrodes (IDEs) consisting of 200 nm Ti and 100 nm Au layers, with a gap of 50 mm on the multiple network ZnO HTs and NTs covered with TiO_2_ NPs gas sensors (total device size: 6.5 × 5 mm^2^, one electrode pad size: 3 x 5 mm^2^). The sensor response to NO_2_ was measured by a PXI chassis system (NI PXIe-1073, National Instruments Inc., Austin, TX, USA). The system chamber was connected to the gas inlet line coming from the mass flow controllers; two mass flow controllers for NO_2_ and air gases were used to control the NO_2_ gas flow rate. A gas exhaust tube was located on the opposite side of the chamber. All the measurements were performed in a closed chamber with the relative humidity (RH) of 50 ± 20% and the temperature of 20.0 ± 10.0 °C (RT), which was maintained at the same humidity and temperature with the normal indoor condition. The electrical resistance of the sensor devices was measured by a source meter (Keithley 2400, Keithley Instruments Inc., Solon, OH, USA) in constant-current operation using a computer-controlled measurement system with the UV illumination (approximately 2.0 W) [21,22]. A custom written LabView program was used, which allowed temperatures and gas-flow rates to be automatically controlled by a computer. Then, we derived the sensor response R as $R\left(\%\right)=R_{g}/R_{a}\times 100,$ where $R_{a}$ is the resistance of the sensor in ambient air, and $R_{g}$ is its resistance under NO_2_ gas.

## 3. Results

**Figure 2:**
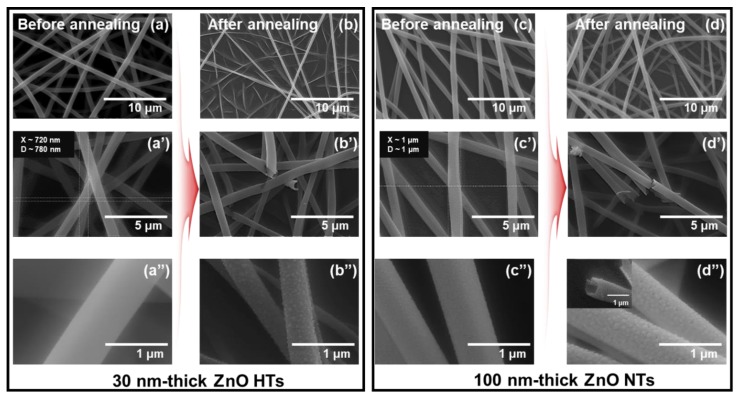
(**a**) FE-SEM image of 30 nm ZnO layer with 10 nm Ti layer (before annealing) on the polyvinylpyrrolidone (PVP) nanowires (NWs), (b) ZnO HTs and NTs covered with TiO_2_ NPs (after annealing), (**c**) FE-SEM image of 100 nm ZnO layer with 10 nm Ti layer (before annealing) on the PVP NWs, and (d) ZnO HTs and NTs covered with TiO_2_ NPs (after annealing). (**a’**)–(**d’**) and (**a’’**)–(**d’’**). High magnification images of (**a**)–(**d**).

Figure 2a,c shows the FE-SEM images of PVP NWs covered with the 30 nm and 100 nm ZnO layer/10 nm Ti layer, respectively. After an 800 °C annealing process, the ZnO HTs (Figure 2b) and NTs (Figure 2d) were obtained; the agglomeration of TiO_2_ NPs occurred because of the atomic migration effect. In addition, the surface area of both ZnO HTs and NTs increased almost two times compared to that of the ZnO layer on the PVP NWs. The average diameters of the PVP NWs covered with 30 nm and 100 nm ZnO layers were 800 nm and 1 μm, respectively, as shown in Figure 2a’,c’. After the annealing process, the average diameters of ZnO HTs and NTs shrank such as Figure 2b’,d’ because of the PVP NWs removal.

**Figure 3:**
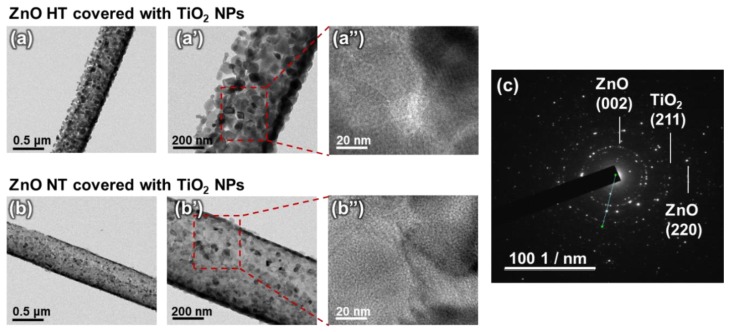
(**a**), (**a’**), (**b**), and (**b’**) Low-magnification bright field (BF) TEM images of ZnO HTs and NTs covered with TiO_2_ NPs. (**a’’**) and (**b’’**) High-resolution TEM images of dotted red rectangular region in the magnified BF TEM images of (**a’**) and (**b’**). (**c**) Selected area electron diffraction (SAED) pattern of the ZnO NT covered with TiO_2_ NPs.

To further investigate the structure properties of ZnO HTs and NTs covered with TiO_2_ NPs, TEM and EDS were measured and analyzed. Figure 3a,a’ and b,b’ show the low-magnification bright field (BF) TEM images of ZnO HT and NT. Figure 3a’’,b’’ show the high-resolution TEM images of dotted red rectangular region in the BF TEM images of Figure 3a’,b’. As shown in these TEM images, the TiO_2_ NPs were covered with the ZnO HT and NT. The corresponding selected area electron diffraction (SAED) pattern in Figure 3c indicated that synthesized nanostructures have polycrystalline crystal structures. Several ring patterns with different diameters were observed, revealing that the nanostructure comprises a polycrystalline structure of ZnO (002), (220) planes, and TiO_2_ NPs corresponding to the (211).

**Figure 4:**
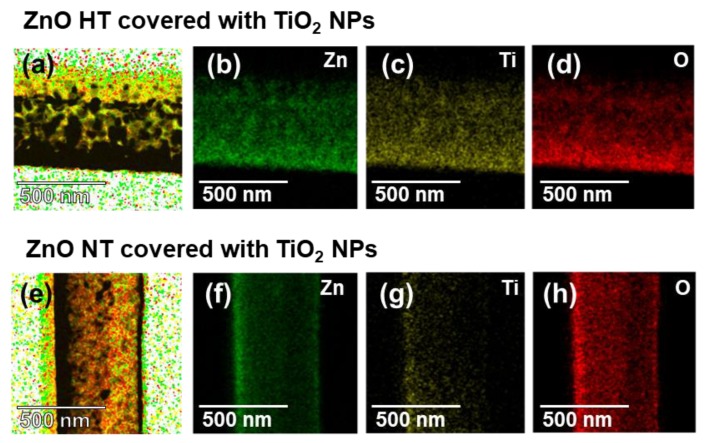
TEM-energy-dispersive spectroscopy (EDS) elemental maps for Zn, Ti, and O in (**a**)–(**d**) ZnO HT and (**e**)–(**h**) NT covered with TiO_2_ NPs.

Figure 4 illustrates the results of the elemental line scanning and mapping measurements of TEM-EDS. The well-defined spatial distributions of O, Zn, and Ti elements in the layers of ZnO HT and NT covered with TiO_2_ NPs were observed as shown in Figure 4a–h. Through the result of EDS analysis, we can confirm that the calculated weight and molar values were predicted to the difference of ZnO layer thickness, as shown in Table 1.

**Figure 5:**
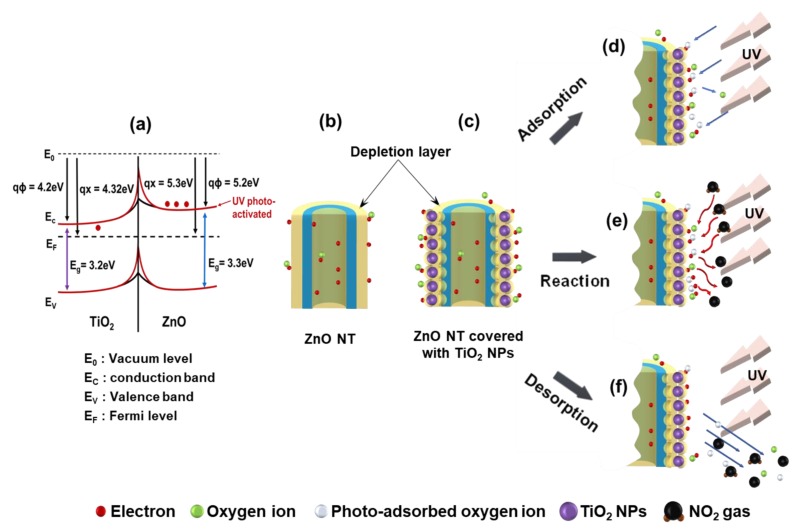
(**a**) Ideal band structure and electron transfer for ZnO and TiO_2_. (**b**)-(**f**) Schematic diagram of possible gas-sensing mechanism of photoactivated ZnO NTs covered with a TiO_2_ NPs gas sensor.

Figure 5 shows the schematic diagram of the possible gas-sensing mechanism of the photoactivated ZnO NTs covered with a TiO_2_ NPs gas sensor. The band bending occurs at the interface between ZnO and TiO_2_. The released photoactivated electrons will flow into the conduction band of the ZnO and TiO_2_ semiconductor because of the higher work function of ZnO (5.3 eV) compared to TiO_2_ (4.2 eV), changing the width and height of the barrier potential at the interfaces, as shown in Figure 5a [23,24,25]. In addition, the width and height of the barrier potential further lower when the UV emitter is illuminated to the active materials of the gas sensor. The remarkable enhancement in the response by UV photoactivated is attributed to the increased change in resistance. Therefore, the change in the ZnO/TiO_2_ interface barrier by UV photoactivation can further contribute to the enhanced NO_2_ gas detection property.

While the sensor detects gas, the detection process of the sensor is divided into adsorption, reaction, and desorption. The sensing mechanism is influenced by the presence of NPs. Since the formation of NPs expanded the depletion layer, the sensing area became large, as shown in Figure 5b,c. Accordingly, the number of electrons that can react with the gas is increased, and the trapped oxygen ions are also increased. Furthermore, UV illumination to the active materials of the gas sensor produces more photoadsorbed oxygen ions. Normally, when air enters the metal oxide semiconductor, oxygen is adsorbed as molecules at low temperature or as the atomic form (O^2-^ and O^-^) at high temperature [24]. The thermal energy at high temperature, which is the cause of the activated oxygen ions, is replaced by the photon energy of the UV emitter. Photon energy can increase the number of charge carriers in the conduction band, improving the surface chemical activity and providing more active areas on the surface (Figure 5d) [26,27]. The larger the surface area, the greater the probability that the gas can react. Gas reactions can be rapidly carried out over large areas (Figure 5e). When the gas injection is completed and the air is injected again, both the reacted gas and oxygen ions are desorbed as shown in Figure 5f.

**Figure 6:**
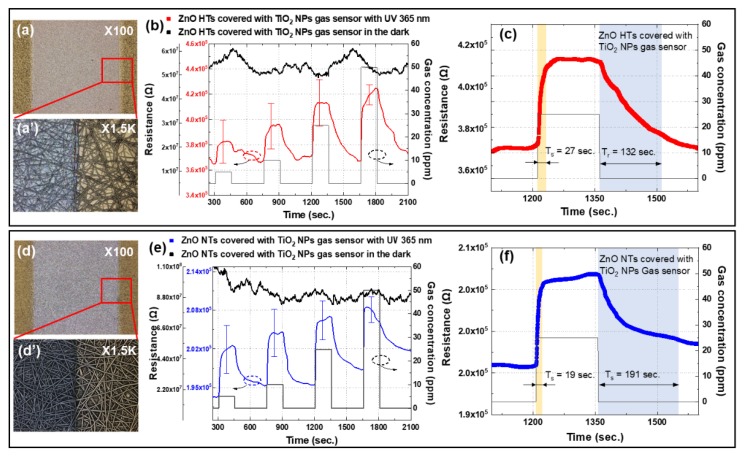
(**a**) Confocal microscopy image of gas sensor consisting of ZnO HTs covered with TiO_2_ NPs, (**a’**) high-magnification image, (**b**) resistance of ZnO HTs covered with TiO_2_ NPs gas sensor in response to different NO_2_ concentrations with and without UV illumination, (**c**) resistance of ZnO HTs covered with TiO_2_ NPs gas sensor to 25 ppm of NO_2_, (d) Confocal microscopy image of gas sensor consisting of ZnO NTs covered with TiO_2_ NPs, (**d’**) high-magnification image, (**e**) resistance of ZnO NTs covered with TiO_2_ NPs gas sensor in response to different NO_2_ concentrations with and without UV illumination, and (**f**) resistance of ZnO NTs covered with TiO_2_ NPs gas sensor to 25 ppm of NO_2_

Figure 6a,d and a’,d’ show the low- and high-magnification confocal microscopy images of the fabricated gas sensors, revealing the multiple network ZnO HTs and NTs covered with TiO_2_ NPs. Compared to Figure 6a’ of ZnO HTs with a TiO_2_ NPs gas sensor, the ZnO NTs with a TiO_2_ NPs gas sensor exhibited a higher density of ZnO structures than the ZnO HTs, as shown in Figure 6d’. Accordingly, the resistance of gas sensors could be detected by the multiple network semiconductor materials.

The resistance variations of the photoactivated RT gas sensors by a UV emitter in response to different NO_2_ gas concentrations are plotted in Figure 6b,e. Sensitivity variations as RH are an important factor in gas sensors; however, it is also very important for a stable NO_2_ gas detection as gas concentrations in normal indoor conditions, repeatedly, as these provided references [28,29,30,31].

ZnO HTs covered with a TiO_2_ NPs gas sensor showed responses of approximately 105%, 108%, 123%, and 116% for 5, 10, 25, and 50 ppm of NO_2_ gas, respectively. While the corresponding response of ZnO NTs covered with a TiO_2_ NPs gas sensor was 104%, 105%, 106%, and 105%, respectively. In Figure 6b,e, the sensitivity changed along with the NO_2_ gas concentrations. A gas sensor fabricated with ZnO NTs had relatively low sensitivity than a gas sensor consisting of ZnO HTs.

This difference can be attributed to the two following reasons. First, ZnO HTs are the structures that the oxygen ions can more easily adsorption and desorption. It means the ZnO HTs have higher oxygen vacancy-related defects than ZnO NTs that are able to generate more active sites for gas molecules, resulting in higher gas sensitivity [32,33,34]. Second, the semiconductor wall thickness is related to the sensitivity of the depletion region [35]. The improvement of sensor response can be attributed to the increase in the depletion layer. In other words, the thinner wall thickness of ZnO HT than NT has a larger depletion layer and higher sensitivity. Accordingly, the gas sensor fabricated with ZnO HTs with TiO_2_ NPs showed better detection results of 1.2 times than the gas sensor consisting of ZnO NTs with TiO_2_ NPs for 25 ppm of NO_2_ gas. This result is relatively similar and has a higher value compared to other ZnO-based RT gas sensors for NO_2_ gas detection [28,36,37].

To confirm the sensing properties of ZnO HTs and NTs gas sensors, the resistance of gas sensors with 25 ppm NO_2_ gas is shown in Figure 6c–f. In Figure 6c, we confirmed that the response and recovery time of gas sensors fabricated with ZnO HTs covered with TiO_2_ NPs were 27 and 132 s. Figure 6f shows the response and recovery time of ZnO NTs covered with a TiO_2_ NPs gas sensor were correspondingly 19 and 191 s. However, the gas sensors did not show any resistance variation at RT without UV illumination. The response time of ZnO HTs covered with TiO_2_ NPs gas sensor is more rapid than that of ZnO NTs covered with TiO_2_ NPs gas sensor, but the recovery speed was reduced. The high surface area and low density of the grain boundaries are the best way to improve the sensitivity of gas sensors [38], which exhibited a dramatic improvement in sensing time for NO_2_ gas. A high density of ZnO NTs could affect the response and recovery time because the influence of intergrain boundaries on the sensor response resistance could become stronger than that of the contact resistance [39,40]. Therefore, the gas sensor with a low density of ZnO HTs could achieve better gas-sensing performance compared to the gas sensor with ZnO NTs. Table 2 clearly shows the sensing properties of gas sensors as gas concentrations. It shows the gas sensor consisted of ZnO HTs has higher gas sensitivity related to gas concentrations than ZnO NTs.

## 4. Conclusions

In conclusion, we fabricated the RT gas sensor for NO_2_ gas detection by ZnO HTs and NTs covered with TiO_2_ NPs by synthesized PVP NWs and ZnO/Ti deposition. The ZnO NTs are able to provide high sensitivity because of their large length-to-diameter ratio and surface-to-volume ratio compared with the other nanostructures. The tube morphology of ZnO HTs and NTs was controlled by the ZnO deposition thickness. In addition, to solve the problem of sensing material stability at high temperature and obtain high energy efficiency, we measured the gas-sensing properties with UV illumination onto the active region of the gas sensor for photoactivation instead of conventional thermal activation by heating. All the measurements were repeatedly performed in a closed chamber with the RH of 50 ± 20% and the temperature of 20.0 ± 10.0 °C (RT), which was maintained at the same humidity and temperature with the normal indoor condition. Sensitivity variations as humidity are an important factor in gas sensors; however, it is also very important for stable NO_2_ gas detection as gas concentrations in normal indoor conditions, repeatedly. Therefore, it will be the aim of future works to determine the RH sensitivity of the sensors to quantify their cross-sensitivity. The width and height of the barrier potential further lower when the UV emitter is illuminated to the active materials of the gas sensor. The remarkable enhancement in the response by UV photoactivation is attributed to the increased change in resistance. In addition, the bended potential barrier between ZnO and TiO_2_ can contribute to the improvement of the NO_2_ gas-sensing properties. Furthermore, the expansion of the depletion region by the TiO_2_ NPs further improved the gas sensitivity. The gas sensor fabricated with ZnO HTs covered with TiO_2_ NPs showed better detection results that were about 1.2 times that of the gas sensor consisting of ZnO NTs with TiO_2_ NPs at the 25 ppm NO_2_ gas. It is because of the thinner and broader depletion region of ZnO HTs compared to the ZnO NTs that higher adsorption and desorption reactions occurred. The ZnO NTs density of the gas sensor could affect the response and recovery time because the influence of intergrain boundary resistance could become stronger than the influence of contact resistance in the sensor. As a result, the detection time of the lower density ZnO HTs was more rapid than that of the higher density ZnO NTs. Therefore, ZnO HTs covered with a TiO_2_ NPs gas sensor can be considered an optimized gas sensor for RT applications. The methodology proposed in this study could be applicable to methods to fabricate RT-operating gas sensors.

## Figures and Tables

**Table 1:** Weight and atomic values of a ZnO HT and a ZnO NT covered with TiO_2_ NPs.

Element	ZnO HT Covered with TiO_2_ NPs		ZnO NT Covered with TiO_2_ NPs	
	Weight (%)	Atomic (%)	Weight (%)	Atomic (%)
**O**	53.96	81.35	38.25	70.81
**Ti**	12.30	6.19	5.00	2.97
**Zn**	33.75	12.46	56.75	26.22
**Totals**	100.00	100.00	100.00	100.00

**Table 2:** Gas-sensing properties of ZnO HTs and ZnO NTs covered with TiO_2_ NPs gas sensors as gas concentrations.

Gas-sensing materials	NO_2_ concentration (ppm)	Response (%)	Response time (s)	Recovery time (s)
**ZnO HTs covered with TiO_2_ NPs**	5	105	26	224
	10	108	43	139
	25	123	27	132
	50	116	59	> 300
**ZnO NTs covered with TiO_2_ NPs**	5	104	36	271
	10	105	18	155
	25	106	19	191
	50	105	11	> 300
